# Study of Intraocular Pressure following Neodynium Yttrium Aluminium Garnet Laser Capsulotomy with the Use of Brimonidine: A Descriptive Cross-sectional Study

**DOI:** 10.31729/jnma.5130

**Published:** 2021-01-31

**Authors:** Laxmi Devi Manandhar, Nanda Gurung, Koshal Shrestha, Binita Bhattarai, Manita Sunam Godar, Rahul Shrestha

**Affiliations:** 1 Department of General Ophthalmology, Lumbini Eye Institute and research centre, Bhairahawa, Nepal; 2 Department of Orthopedics, Lumbini City Hospital and Medical sciences, Butwal, Nepal

**Keywords:** *brimonidine*, *intraocular pressure*, *Nd:YAG laser capsulotomy*

## Abstract

**Introduction:**

Posterior capsular opacification is a common complication after cataract surgery. Neodynium Yttrium Aluminium Garnet laser capsulotomy is still the preferred treatment for posterior capsular opacification. This study was done to determine the use of Brimonidine eye drop in preventing the rise of intraocular pressure post-Neodynium Yttrium Aluminium Garnet laser capsulotomy.

**Methods:**

A descriptive cross-sectional study was conducted in Lumbini eye institute and research center, Bhairahawa, Nepal, in 200 eyes with posterior capsular opacification using Brimonidine from Feb 1, 2019, to July 30, 2019. The Institutional Review Committee approved the study with approval number 0237. A convenient sampling method was used. Pre-capsulotomy best-corrected visual acuity, slit-lamp examination of the anterior segment, and dilated fundus examination were done. Intraocular pressure was measured with Goldmann Applanation Tonometer. Post capsulotomy patients were evaluated after one hour, two hours, and two weeks for intraocular pressure and any complications. The statistical analysis was done using Statistical Package of Social Sciences version 20.0 statistical analysis software. The descriptive statistical analysis of the study was done after the collection of the data.

**Results:**

Mean age of patients at presentation was 61.61 ±SD 1.09. The mean intraocular pressure following Neodynium Yttrium aluminum garnet laser capsulotomy using brimonidine at 1 hour was 12.73±3.3 mmHg. and two hours was 11.98±3.2 mmHg. The mean energy per pulse was 2.3 ±SD 0.3 mJ. The mean duration of posterior capsular opacification from cataract surgery was 22.28 weeks.

**Conclusions:**

Neodynium Yttrium Aluminium Garnet laser capsulotomy had lower intraocular pressure after the Brimonidine eye drop procedure. The maximum mean reduction in intraocular pressure was observed after two hours.

## INTRODUCTION

Posterior capsular opacification (PCO) is a frequent complication after cataract surgery.^1^ The standard treatment for PCO is Neodynium Yttrium Aluminium Garnet (Nd:YAG) laser posterior capsulotomy, with a more than 95% success rate.^2^ A transient rise of intraocular pressure (IOP) is the most common complication following laser capsulotomy, which may occur in 15 to 36% of patients who receive no prophylactic treatment.^3,4^ It is due to debris deposition in the trabecular meshwork, pupillary block, and inflammatory swelling of the ciliary body or iris root associated with angle closure.^5^

Nd:YAG capsulotomy is a common procedure done in the Lumbini eye institute. So, the study of IOP after YAG capsulotomy may provide a better concept on providing better services to patients.

This study was done to study the use of Brimonidine eye drop in preventing the rise of IOP post-Nd:YAG laser capsulotomy in a tertiary care center.

## METHODS

A descriptive cross-sectional study was carried out at the outpatient department (OPD) of Lumbini eye institute and research center, Bhairahawa, Nepal, within 6 months from Feb 1, 2019 to July 30, 2019. The ethical approval committee has approved this research with approval number 0237 and the Institutional review board.

The sample size was calculated using the formula,

$$\begin{array}{ll} \text{n} & ={\text{Z}}^{\text{2}}\text{p}\times \text{q}/{\text{e}}^{\text{2}}\\ & ={\left(1.96\right)}^{\text{2}}\times 0.5\times 0.5/0.07^{\text{2}}\\ & =196 \end{array}$$

Where,

n = required sample size
Z = 1.96 at 95% confidence interval
p = prevalence 50% for maximum sample size
q = 1-p
e = margin of error, 7%

Hence, we took a sample of 200 in our study by convenient sampling method.

Inclusion criteria for enrolment were patients aged more than 20 years who underwent uneventful cataract surgery with posterior chamber intraocular lens (PCIOL) implantation with PCO. Patients diagnosed with diabetic retinopathy, retinal detachment, corneal disease, glaucoma, post trabeculectomy, uncooperative patient, and those who cannot come for follow-up and patients having PCO of fewer than three months were excluded from the study. 200 eyes who underwent Nd:YAG laser capsulotomy with the use of Brimonidine eye drop were randomly selected. Informed consent was obtained from the patients enrolled in the study.

**Before performing Nd:** YAG laser capsulotomy, a detailed history was taken, and a complete ocular examination was performed, including assessment of visual acuity (VA) using Snellen's vision. Slit-lamp examination of the anterior segment was done along with the status of PCIOL and PCO grading, tonometry with Goldman applanation tonometer (GAT), and dilated fundus examination. Grading of PCO was done according to The Madurai intraocular lens study IV: posterior capsule opacification.^6^ A grading of I to III was used to reflect the degree of opacification. The statistical analysis was done using SPSS (Statistical Package of Social Sciences) Version 20.0 Statistical Analysis Software. The descriptive statistical analysis of the study was done after the collection of the data.

**Procedure:**

Nd: YAG laser capsulotomy was performed in OPD as a daycare procedure by an experienced ophthalmologist. Topical Brimonidine eye drop was used in all patients one hour prior to the procedure. Post-procedure topical diclofenac four times per day for one week was prescribed to all patients. Patients were re-evaluated at one hour, two hours after the procedure and again followed up at two weeks. Examination at each subsequent evaluation included measurement of IOP using GAT, anterior chamber examination, intraocular lens (IOL), vitreous, and retina status. Any complications were noted during each follow-up. Topical Brimonidine eye drop was started in eyes with raised IOP of more than 30mmHg.

## RESULTS

Just monitoring the data of brimonidine used would be sufficient. No comparison group study is required. A total of 200 eyes of 200 patients were studied. The mean age of patients at presentation was 61.12 years ±SD 1.29. There were 85 (42.5%) male patients and 115 (57.5%) female patients. The right eye is affected more than the left eye. In this study, the mean energy per shot was 2.26mJ±SD 0.3 (Table 1). The mean IOP at one hour was 12.73±3.3 and the two hours was 11.98±3.215.47±5.0 mmHg. An increase in IOP of more than 30 mmHg was seen in 2 patients in two hours. They were managed with topical brimonidine for one week. An increase in IOP more than 5 mmHg after Nd:YAG laser posterior capsulotomy was termed as raised post-procedure IOP. Visual acuity was improved in all patients (Table 2).

**Table 1:** Pre-procedure characteristics of patients.

Patients Characteristics	Mean (n=200)	Percentage (%)
Age in years	61.6 ±SD1.09	
Sex Male		85 ( 42.5)
Female		115 (57.5)
Duration of PCO from cataract surgery in weeks	22.28±SD 19.46	
Energy per shots	2.26mj ±SD 0.3	
Number of shots	19.93 SD±8.3	
Right eye		110 (55)
Left eye		90 (45)

**Table 2:** Distribution of Mean IOP (mmHg) and Visual acuity (VA) at each time interval.

	IOP mmHg		VA logmar n=200	
Time intervals	Mean	SD	Mean	SD
Pre capsulotomy	13.36	3.08	0.84	0.33
1 hour post capsulotomy	12.73	3.3	0.53	0.30
2 hours post capsulotomy	11.98	3.2	0.53	0.30
2 weeks post capsulotomy	13.08	2.9	0.33	0.22

PCIOL pitting 17.5% was the most common complication observed in our study. Iritis, hyphema, ruptured anterior hyaloid were also observed in our study. All patients were successfully treated. One patient who had ruptured anterior hyaloid face was treated with anterior vitrectomy (Table 3). Only two patients had an IOP rise of more than 30 mmHg. Those patients required topical anti-glaucoma (brimonidine) for 1 week.

**Table 3:** Complications of YAG laser Capsulotomy.

Complications	No. of patients n (%)
None	122 (61.0)
PCIOL pitting	35 (17.5)
IOP rise	2 (1.0)
Iritis	34 (17.0)
Hyphema	3(1.5)
Vitreous in anterior chamber	1 (0.5)
PCIOL subluxation	3(1.5)
Total	200 (100)

## DISCUSSION

IOP increase after Nd: YAG laser capsulotomy is a complication reported at various intervals. 59-67% of patients showed IOP increment of at least 10 mmHg following Nd:YAG laser capsulotomy in the absence of anti-glaucoma prophylaxis.^2^ Brimonidine has been tried to decrease the rise in IOP after YAG laser capsulotomy by several researchers.^7,8^

In our study, the mean age at presentation was 61 yrs. This finding corresponds to the study done by Parajuli et al. and Shetty et al., which also found mean age between 50 to 70 years.^2,9^ This finding is due to the elderly being the most common age group undergoing cataract surgery.

A study done by Singhal et al. from India reported that raised IOP occurred after four hours of YAG laser capsulotomy where fall in 1-5 mmHg of IOP was seen with the use of brimonidine.^7^ This study also showed similar findings, but the fall in IOP was maximum in two hours. Similar to this study, Seong also found IOP decreased from the baseline by the third postoperative hour.^10^

Brimonidine has been proven effective to counteract the increase in IOP following Nd-YAG laser capsulotomy. Our study found that a decrease in IOP with Brimonidine after 2 hrs of YAG laser capsulotomy was by 1.4 mmHg. A study led by Yeom documented that Brimonidine 0.2% and Brimonidine Purite 0.15% have similar efficacy in the prevention of IOP elevation after Nd:YAG laser posterior capsulotomy with a decrease in IOP from baseline ranged from 2.3 to 2.7 mmHg.^8^

A study in India in 2015 showed that those patients who received more than 40 shots had a significant rise in IOP and required treatment in the form of anti-glaucoma medications immediately after the procedure for one-week post-procedure.^9^ Our finding was almost similar to this study. This may be explained by the energy delivered to the eye, which creates the shock waves that disturb the ciliary body or causes the neurohumoral increase in IOP.

Our study's limitation was the short duration of follow up so long-term complications could not be studied. Similarly, this study was conducted in a single tertiary care center, due to which the results cannot be generalized to all the tertiary care centers of Nepal.

## CONCLUSIONS

Nd:YAG laser posterior capsulotomy is an effective and safe method for the management of posterior capsular opacification. Pre-capsulotomy Brimonidine is highly effective in controlling IOP after Nd:YAG laser posterior capsulotomy.
